# An Enhanced Reservation-Based MAC Protocol for IEEE 802.15.4 Networks

**DOI:** 10.3390/s110403852

**Published:** 2011-03-30

**Authors:** José A. Afonso, Helder D. Silva, Pedro Macedo, Luis A. Rocha

**Affiliations:** 1 Department of Industrial Electronics, University of Minho, Campus of Azurém, 4800-058 Guimarães, Portugal; E-Mails: dsilva@dei.uminho.pt (H.D.S.); pmacedo@dei.uminho.pt (P.M.); 2 IPC/I3N, University of Minho, Campus of Azurém, 4800-058 Guimarães, Portugal; E-Mail: lrocha@dei.uminho.pt

**Keywords:** wireless sensor networks, medium access control, quality of service

## Abstract

The IEEE 802.15.4 Medium Access Control (MAC) protocol is an enabling standard for wireless sensor networks. In order to support applications requiring dedicated bandwidth or bounded delay, it provides a reservation-based scheme named Guaranteed Time Slot (GTS). However, the GTS scheme presents some drawbacks, such as inefficient bandwidth utilization and support to a maximum of only seven devices. This paper presents eLPRT (enhanced Low Power Real Time), a new reservation-based MAC protocol that introduces several performance enhancing features in comparison to the GTS scheme. This MAC protocol builds on top of LPRT (Low Power Real Time) and includes various mechanisms designed to increase data transmission reliability against channel errors, improve bandwidth utilization and increase the number of supported devices. A motion capture system based on inertial and magnetic sensors has been used to validate the protocol. The effectiveness of the performance enhancements introduced by each of the new features is demonstrated through the provision of both simulation and experimental results.

## 1. Introduction

IEEE 802.15.4 [1,2] is a standard designed to address the needs of low power consumption, low data rate and low cost wireless networking applications. It has been applied in areas such as industrial monitoring, personal healthcare and residential automation. As the demand for real-time characteristics increases, quality of service (QoS) support becomes an important issue in these areas. Due to collisions, the mandatory medium access control (MAC) protocol provided by the IEEE 802.15.4 standard (contention-based CSMA/CA scheme) is not tailored to provide QoS support required by real-time applications, especially under high loads. The standard also provides an optional reservation-based Guaranteed Time Slot (GTS) scheme intended to support devices requiring dedicated bandwidth or low latency transmission. However, the active portion of the superframe is divided into only 16 slots, and since a device has to allocate at least a full GTS slot, bandwidth is wasted when slots are used partially. Moreover, only seven GTS allocations are allowed, according to the standard specifications in [1].

Some proposals have been presented in literature to increase bandwidth utilization of the GTS scheme. In [3], authors propose the use of the backoff period defined by the standard, which is smaller than the slot duration, as the allocation unit, in order to reduce the waste of bandwidth. In addition, the slot allocation information, carried by the beacon, is renewed at every superframe, allowing nodes to use different periodic cycles for dedicated transfer. However, the constant renewing of the allocation information coupled with the use of the backoff period (which requires more bits to encode the position and length of the allocation) tend to increase the beacon length significantly. This leads to an increase in the beacon’s vulnerability to channel errors, thus reducing the packet delivery ratio (allocated transmissions cannot be performed when the beacon is lost).

The i-GAME mechanism proposed in [4] improves bandwidth utilization by allowing several nodes to share the same set of GTS slots. However, the method used by the PAN coordinator to enforce the sharing order of the GTS slots among the nodes remains unanswered. Moreover, this mechanism also requires the requesting nodes to identify traffic specifications and delay requirements of its flows, which tends to increase the complexity of the implementation.

The scheme proposed in [5] divides the Contention Free Period (CFP) into 16 equally sized slots, in contrast with the division of the whole superframe as in GTS. This scheme is able to keep the format of the GTS descriptor intact, changing only the way devices interpret the GTS allocation fields. One drawback of this approach is that the slot duration becomes dependent of the CFP length, and therefore current allocations become inconsistent when the length of the CFP needs to change in order to accept new allocations. Moreover, the number of slots provided remains small.

The eLPRT protocol described in this paper presents several features designed to enhance the performance in comparison to the GTS scheme of IEEE 802.15.4. Besides improving bandwidth utilization and increasing the number of supported devices, the eLPRT protocol introduces various mechanisms designed to increase the packet delivery ratio in the presence of channel errors. Most of the proposed features are independent of each other and can be adopted separately in the scope of future versions of the IEEE 802.15.4.

The eLPRT protocol was implemented and tested in the development context of a motion capture system based on multiple wireless sensor nodes that integrate inertial and magnetic sensors [6,7]. The system presents a modular and versatile architecture where the wireless sensor nodes are placed only in the user’s body segments that need to be monitored. Another advantage of this system is that it can be used to monitor several users in the same area at the same time using a single wireless network; hence, maximization of the number of modules supported by the wireless network is desirable.

The traffic generated by the developed motion capture system is used in this paper to provide the application scenario where the performance of the eLPRT protocol and the IEEE 802.15.4 schemes are compared. The monitoring of some data-intensive biomedical signals, such as ECG, is an example of another application area with similar traffic characteristics and QoS requirements [8].

New mechanisms to improve protocol performance, namely a reallocation mechanism and a frequency hopping mechanism are enhancements introduced by eLPRT to the original LPRT protocol. This paper describes and analyzes the main features of the eLPRT protocol, comparing each one to the corresponding functionality provided by the GTS scheme.

This paper is organized as follows. The next section presents an overview of the IEEE 802.15.4 standard. The features provided by the eLPRT protocol are described in Section 3. Section 4 describes the input parameters and simulation models used to evaluate the performance of the protocols. Section 5 demonstrates the inadequacy of the IEEE 802.15.4 unslotted CSMA/CA scheme relative to the motion capture application scenario. Section 6 presents an analysis of the eLPRT protocol features, comparing each one to the corresponding functionality provided by the GTS scheme. Section 7 describes the implementation of the eLPRT protocol in the context of the developed motion capture system and provides experimental results. Finally, Section 8 presents the conclusions.

## 2. IEEE 802.15.4

The IEEE 802.15.4 [1] is a standard that specifies the physical (PHY) and medium access control (MAC) layers for low-rate wireless personal area networks (LR-WPANs). This standard targets typical requirements of wireless sensor networks such as low power consumption, low data rate and low cost. The physical layer specifies three operating frequency bands, providing a single channel in the 868 MHz band, 10 channels in the 915 MHz band and 16 channels in the 2.4 GHz band. Available data rates vary from 20 kbps to 250 kbps depending on the frequency band.

The MAC layer provides two different modes of operation: non beacon-enabled mode and beacon-enabled mode. The non beacon-enabled mode relies on an unslotted CSMA/CA mechanism, while the beacon-enabled mode provides a slotted CSMA/CA mechanism and an optional reservation-based mechanism called guaranteed time slot (GTS).

**Figure 1.**
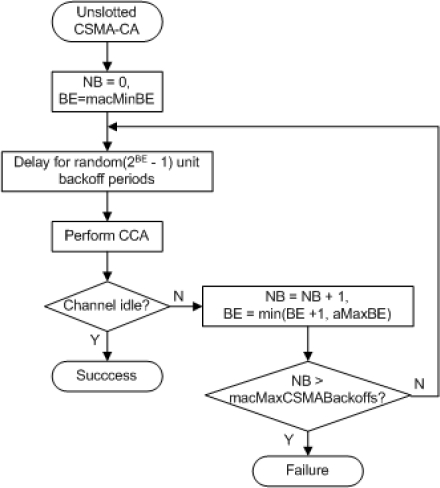
Unslotted CSMA/CA algorithm.

The unslotted CSMA/CA algorithm [1] is represented in Figure 1. Before accessing the channel, the device must wait for a random backoff interval defined in the range from 0 to (2*^BE^* − 1) backoff periods. The backoff exponent (*BE*) initially takes the value *macMinBE* and one backoff period is equal to *aUnitBackoffPeriod* symbols. After waiting for a random interval, if the clear channel assessment (CCA) function indicates that the channel is idle, the device starts its transmission after a turnaround time delay, which is the time necessary for the radio transceiver to switch from receive state to the transmit state. If the channel is busy, the device defers its transmission and increments the number of transmission attempts for the current packet (*NB*). *BE* is also incremented if it has not reached its maximum value, *aMaxBE*. If the maximum number of transmission attempts, *macMaxCSMAbackoffs*, was not reached, a new backoff interval is determined; otherwise, the algorithm declares a channel access failure. The unslotted CSMA/CA parameters are specified in Table 1.

**Figure 2.**
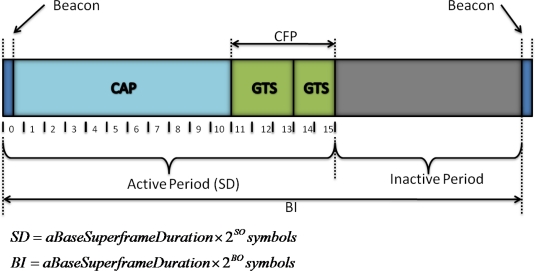
Example of the superframe structure of IEEE 802.15.4.

The beacon-enabled mode imposes the use of a superframe structure delimited by two consecutive beacons, being composed by an active and an inactive portion. An example of the superframe structure is presented in Figure 2. The attribute *macSuperframeOrder* (*SO*) defines the duration of the active period, which is called Superframe Duration (*SD*), while the attribute *macBeaconOrder* (*BO*) defines the Beacon Interval (*BI*). The minimum length of the superframe (*aBaseSuperframeDuration*) corresponds to 960 symbols and 0 ≤ *SO* ≤ *BO* ≤ 14. The active portion of the superframe is composed by a beacon frame, a Contention Access Period (CAP) and a Contention Free Period (CFP). The slotted CSMA/CA scheme is used during the CAP and the GTS scheme is used during the CFP. The minimum length of the CAP is given by *aMinCAPLength* = 440 symbols.

The GTS scheme aims to provide some level of quality of service (QoS) support for applications requiring bounded delay or bandwidth guarantees. However, only a maximum of seven GTS allocations are allowed. The active portion of the superframe is divided into 16 slots and a GTS allocation can take an integer number of these slots. In the example of Figure 2, a GTS allocation of three slots and another of two slots are shown. The process of slot allocation in the CFP starts with the transmission of a GTS request command, from the device to the PAN coordinator. The request indicates the GTS Characteristics, which are composed by the GTS Length (number of slots being requested); the GTS Direction: ‘0’ for uplink (sending data) or ‘1’ for downlink (receiving data); and the Characteristic Type: ‘1’ for allocation or ‘0’ for deallocation. On receipt of a GTS allocation request, the PAN coordinator sends back an acknowledgement frame.

If there are resources available in the superframe, the PAN coordinator announces the allocation to the device through the inclusion of a GTS descriptor in the beacon. The GTS descriptor has a length of 3 bytes and is composed by the Device Short Address (16 bits), the GTS Start Slot (4 bits) and the GTS Length (4 bits). The GTS descriptor remains in the beacon for *aGTSDescPersistenceTime* (= 4) superframes, after which it is removed. If a device misses the beacon in the beginning of a superframe, it must not use its allocated GTS slots until it receives a subsequent beacon correctly.

The deallocation of a GTS may result in the superframe becoming fragmented. In this case, the PAN coordinator must perform a GTS reallocation to remove the gap in the CFP in order to maximize the CAP length.

## 3. The eLPRT Protocol

The eLPRT is an enhanced version of the LPRT protocol described in [9], and therefore it inherits most of the characteristics of the latter. The eLPRT introduces new mechanisms to improve the performance and uses protocol message formats similar to the ones used by the IEEE 802.15.4. In the original LPRT protocol, the allocation information announced in the beacon frame is valid only for the respective superframe, while in the eLPRT protocol and GTS schemes, the allocation information is valid for successive superframes until it is changed. In order to preserve clarity to the reader, the following description of eLPRT also includes previous LPRT features, and focuses on the differences to the GTS scheme.

The eLPRT MAC protocol presents a superframe structure similar to the one defined by the IEEE 802.15.4, but introduces several new features designed to increase the number of supported devices, improve bandwidth utilization and increase the robustness against channel errors. Each of these features enhances one aspect of the performance in comparison with the GTS scheme, and most of them can be adopted independently from each other.

A basic feature of the eLPRT and LPRT protocols is the division of the superframe into a much larger number of slots (500, in the current implementation) than the IEEE 802.15.4 (16 slots). This feature increases significantly the granularity of slot allocation in the CFP, avoiding the waste of bandwidth and, consequently, contributing to increase the throughput efficiency and the number of supported nodes. The price of this increased granularity is reflected in terms of tighter synchronization bounds that need to be respected by both coordinator and node devices. The increased granularity also increases the number of bits required to encode the allocation length and start slot.

Another feature of the eLPRT protocol is the provision of a larger set of options for the superframe period, to closely match the packet generation interval imposed by the application.

Similarly to the GTS scheme, in order to allocate/deallocate slots in the CFP, the sensor node sends an Allocation Request command containing the Allocation Length, the Allocation Direction and the Allocation Type (allocation or deallocation). While in the GTS scheme the sensor node receives an ACK frame as response, in the eLPRT and LPRT protocols the node receives an Allocation Response command containing an Allocation Identifier (AID), which is assigned dynamically to the node during allocation and released in deallocation. The AID is shorter than the Device Short Address (16 bits) that is used by the IEEE 802.15.4. In the current implementation, the length of the AID was set to 6 bits, which is enough to support the traffic of up to 64 nodes in the CFP and is significantly greater than the seven nodes allowed by the GTS scheme. The eLPRT allocation descriptor is composed by 6 bits for the AID, 9 bits for the Start Slot and 9 bits for the Allocation Length. Thanks to the shorter AID, the length of the eLPRT allocation descriptor is the same of the corresponding GTS descriptor of the IEEE 802.15.4 (3 bytes), even though the reference to a slot requires more bits in the eLPRT protocol (9 bits) than in the GTS scheme (4 bits).

Another feature of the eLPRT and LPRT protocols is the inclusion of an ACK bitmap field in the beacon, which provides the acknowledgment of uplink transmissions made in the CFP of the previous superframe. This feature eliminates the protocol overhead associated with the reception of individual ACK frames for each uplink data packet. The correspondence between each uplink transmission and the bits of the ACK bitmap is made using the AID of the corresponding node. For downlink transmissions, the conventional ACK frame is used.

In the GTS scheme of the IEEE 802.15.4, all allocations take effect from the moment they are announced in the beacon. In the event of a GTS reallocation, the slots previously allocated to a node can be reallocated to another one. Therefore, whenever a node misses the beacon at the beginning of a superframe, it must not transmit in the allocated slots of that superframe, since its allocation could have changed. The eLPRT protocol introduces a new reallocation mechanism that enables the use of slots allocated for transmission in a superframe when the corresponding beacon is lost. This mechanism works through the inclusion of a reallocation counter (RC) field in the beacon that normally takes the value zero (0). Whenever a reallocation is necessary, the RC field is changed to the integer value *N_RC_* (15 in the current implementation). During consecutive beacons, the value of the RC field is decremented, until it reaches zero again. While *N_RC_* > 0, all changes in allocations are announced, through the inclusion of the corresponding updated allocation descriptors in the beacon; however, the new allocations only take effect when the value of the RC field returns to zero. This mechanism ensures that a node can use its allocated slots even when it misses up to *N_RC_* consecutive beacons.

**Figure 3.**
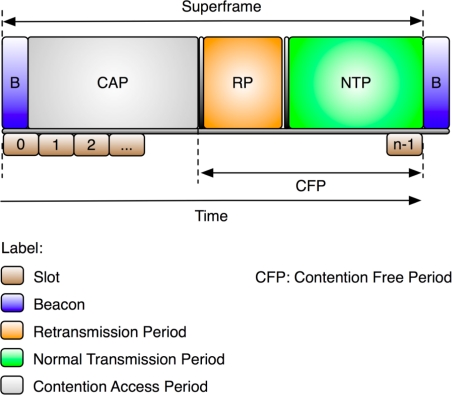
Superframe structure of the eLPRT protocol.

An additional feature of the eLPRT and LPRT protocols is the provision of a contention-free retransmission mechanism. This mechanism provides automatic allocations, on demand, of supplementary slots in a superframe for retransmission of data packets whose original transmission failed in the previous superframe. These supplementary slots form an extra reserved period in the superframe referred as Retransmission Period (RP). As shown in Figure 3, the RP is placed after the Contention Access Period (CAP) and before the period reserved for the original transmissions, which is named Normal Transmission Period (NTP). The eLPRT allocation descriptor for retransmissions requires only 2 bytes, because the allocation length does not need to be announced, since it is the same of the original transmission. The goal of the retransmission mechanism is to increase the reliability in case of channel errors while avoiding collisions. With this mechanism, extra bandwidth is automatically allocated to isochronous traffic connections, in detriment of asynchronous traffic transmitted in the CAP, in order to better fulfill QoS requirements for scheduled allocations.

The eLPRT protocol also introduces a simple frequency hopping mechanism to deal with interference, especially from IEEE 802.11 networks. The operating frequency changes from superframe to superframe, according to Equation (1), where *i_s_* represents the index of the superframe and *n_j_* is the channel jump adopted. This jump has to be an odd number to ensure that the network jumps through all 16 available channels from the 2.4 GHz frequency band. Since an IEEE 802.11 channel occupies the equivalent to four IEEE 802.15.4 channels, the value of *n_j_* was set to 5 in the current implementation. This ensures that the network does not suffer the interference of an IEEE 802.11 channel during two consecutive superframes. In combination with the retransmission mechanism, the frequency hopping mechanism enables the exploration of frequency diversity to recover from transmission errors:

(1) $$\mathrm{Ch}\left(i_{s}\right)=11+\left(n_{j}\times \mathrm{Ch}\left(i_{s}-1\right)\right)\;\mathrm{mod}\;16$$

## 4. Evaluation Scenario

### 4.1. Traffic, Network and Device Parameters

In this paper, the developed motion capture system [7] is used to provide traffic parameters for performance evaluation of the eLPRT protocol and comparison with the unslotted CSMA/CA and GTS schemes of the IEEE 802.15.4.

Typical motion capture applications, such as character animation, require a frame rate of 30 fps, which means that the sensors have to be sampled at 30 Hz. If the interval between data packets is set to 100 ms, each data packet will carry three samples from each sensor. A smaller interval could be chosen, but it would tend to decrease the number of supported nodes, because the payload length of the data packet would decrease, increasing the protocol overhead.

Each sensor node contains six sensors (three accelerometers and three magnetometers) which are sampled with a resolution of 12 bits. To save space, each two 12-bit samples are compressed into 3 bytes. Each data packet also carries a sample of the battery voltage in 2 bytes. Therefore, the payload length required to carry all these samples is 29 bytes.

The MAC header and trailer fields occupy a total of 11 bytes and the physical layer of the IEEE 802.15.4 requires another 6 bytes. Therefore, the length of data packets, considering the payload, the MAC overhead and the PHY overhead is 46 bytes.

The evaluation is based on parameter values specified by IEEE 802.15.4 standard for the physical layer operating in the 2.4 GHz frequency band. The standard specifies, for this band, a symbol period (SP) of 16 μs, bit rate of 250 kbps, a turnaround time of 192 μs (*aTurnaroundTime* = 12 symbols), minimum superframe period of 15.46 ms and minimum CAP length of 7.04 ms. Other relevant parameters were previously presented in Section 2.

The parameters of the CC2430 [10], which was the device used in the implementation of the eLPRT protocol, were used to provide the simulation results concerning the energy consumption of the nodes due to the operation of the MAC protocols. The CC2430 specifies a current consumption of 26.7 mA in RX mode, 26.9 mA in TX mode (0 dBm) and 190 μA in the used sleep mode (power mode 1).

### 4.2. Simulation Models

OMNeT++, an open-source, modular, component-based C++ simulation library and framework [11], was used to implement the simulation models of the unslotted CSMA/CA scheme of IEEE 802.15.4 and variants of the eLPRT protocol. Each simulation run ended after the base station (PAN coordinator) received 100,000 data packets from the nodes.

The Gilbert-Elliot model [12] was used to model the occurrence of burst errors, which are typical in wireless channels. The model considers a channel alternation between a good state with low bit error rate (*BER_good_*) and a bad state, with high bit error rate (*BER_bad_*), with mean dwelling time *T_good_* for the good state and *T_bad_* for the bad state. The values of the parameters used in simulations, unless otherwise stated, are presented in Table 2. The chosen values are intended to model fast fading, which typically occurs on timescales of milliseconds to tens of milliseconds [13]. The channel state for the different nodes was made symmetrical and independent, which means that at any moment the channel for some nodes can be in the bad state while for others it can be in the good state.

## 5. Analysis of the Unslotted CSMA/CA Scheme of the IEEE 802.15.4

This section analyzes the performance of the unslotted CSMA/CA mechanism of IEEE 802.15.4, considering its use to carry traffic generated by the motion capture system. The first data packet from a sensor node is generated randomly in the interval between zero and 100 ms, since there is no time coordination between the nodes. After that, the sensor node generates data packets periodically with an interval of 100 ms. The parameters of the unslotted CSMA/CA algorithm were provided in Table 1. The interval between the data packet and the ACK frame is 192 μs, since *t_ack_* = *aTurnAroundTime*.

The results presented in this section were obtained under favorable conditions: an error-free channel and no hidden nodes. Four operation modes were considered: do not retransmit if the transmission fails (Without ACK–0 Ret); up to one retransmission attempt per data packet (1 Ret); up to three retransmission attempts per data packet (3 Ret); and up to seven retransmission attempts per data packet (7 Ret). In this simulation scenario, a transmission can only fail due to collision with a packet transmitted by another node in the same network or due to failure to access the channel (which occurs when the node detects a busy channel during *macMaxCSMAbackoffs* attempts).

**Figure 4.**
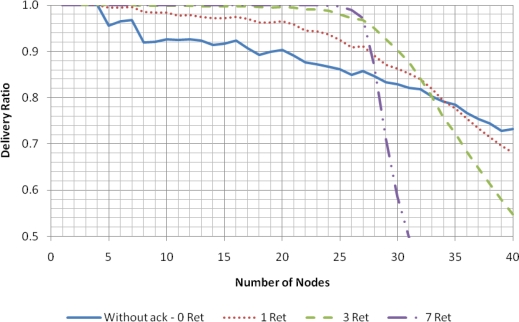
Delivery ratio for the unslotted CSMA/CA scheme with error-free channel.

Figure 4 presents the delivery ratio as a function of the number of sensor nodes in the network. As the results for the mode without retransmissions show, transmissions start to fail with as low as five nodes and, consequently, the delivery ratio decreases as the number of nodes increases. The retransmissions allow the recovery from failures, up to a certain point. When the number of nodes is relatively small, the more retransmission attempts, the better the delivery ratio. However, as the number of nodes increases, the traffic load also increases, increasing the collisions, as well as the channel access failures. Since retransmissions contribute to aggravate the situation, the delivery ratio starts to collapse after a certain point, a phenomenon that is more pronounced when more retransmissions are allowed. In the considered scenario, the unslotted CSMA/CA protocol can support up to 25 nodes at a delivery rate near 100% (with a maximum of seven retransmission attempts).

**Figure 5.**
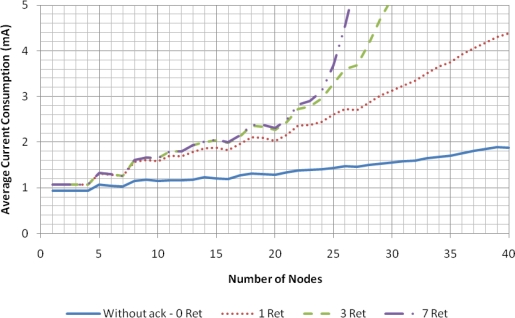
Average current consumption for the unslotted CSMA/CA scheme.

The backoff process and retransmissions also have a significant impact in current consumption of the nodes, as shown in Figure 5, which uses the CC2430 current parameters presented in Section 4.

## 6. Analysis of the eLPRT Protocol

This section analyzes the main features of the eLPRT protocol and compares each one to the corresponding functionality provided by the GTS scheme. In the successive analysis of each particular feature, previously analyzed features are already incorporated in the protocols.

### 6.1. Choice of the Superframe Period

In the 2.4 GHz band, the minimum superframe period defined by the IEEE 802.15.4 standard (according to Figure 2, with *SO* = 0) is 15.46 ms, with the closest allowed superframe periods around 100 ms being 61.44 ms (*SO* = 2) and 122.88 ms (*SO* = 3). The sampling rate (*f_s_*) required by the application scenario (30 Hz) corresponds to a sampling period of 33.33 ms. However, neither of these two superframe periods is multiple of the sampling period, so the number of samples per packet and, consequently, the packet length, vary, as shown in Table 3. Another example where the sampling rate is 10 Hz is also presented. This analysis assumes that the first sample is generated at *t* = 0, which also corresponds to scheduled time for the first transmission.

The eLPRT protocol uses 8 bits to encode the superframe period, allowing 256 options in comparison with the 15 options provided by the GTS scheme. It also provides the superframe period of 100 ms, which means that all packets carry the same number of samples in this case, except for the first one. To allow a fair comparison of the other features, the following results assume that both the eLPRT protocol and the GTS scheme are using a superframe period of 100 ms.

### 6.2. The ACK Bitmap Mechanism

The data packet in the evaluation scenario has a length of 46 bytes, so the corresponding transmission time (*T_data_*) is 1,472 μs. The length of the ACK frame is 11 bytes, which corresponds to a transmission time (*T_ack_*) of 352 μs. Since the interval between the data packet and the ACK (*t_ack_*) is 192 μs, the overhead introduced by the ACK (*O_ack_*) is 37%, according to Equation (2). The ACK bitmap mechanism of the eLPRT protocol eliminates this overhead through the replacement of the ACK frame by a single ACK bit in the following beacon, which results in an increase in the network throughput in the same proportion, as well as a decrease in the node energy consumption:

(2) $$O_{\mathrm{ack}}=\frac{T_{\mathrm{ack}}+t_{\mathrm{ack}}}{T_{\mathrm{data}}}$$

### 6.3. Fine Grained Allocation of Slots

The GTS scheme provides 16 slots, so the slot duration (*T_slot_*) for a superframe period of 100 ms is 6.25 ms. Since the transmission time of the data packet (*T_data_*) is 1.472 ms, the efficiency in the utilization of the allocated time is only 23.6%. The eLPRT provides 500 slots, which means that *T_slot_* = 0.2 ms for the same superframe period. The number of slots required to accommodate the data packet (*N_s_*) is 8, according to Equation (3); therefore, the allocated time is 1.6 ms, which means that the efficiency in this case is 92%, which represents a large improvement over the GTS scheme:

(3) Ns=ceil (TdataTslot)

The minimum duration of the CAP specified by the IEEE 802.15.4 standard for the 2.4 GHz band is 7.04 ms, while the time required to transmit a maximum length beacon is 4.26 ms and therefore the maximum duration of the CFP (*CFP_max_*), obtained from the subtraction of these two values from the superframe period, is 88.7 ms. If we assume that one additional slot is used as guard time between transmissions of the sensor nodes, each data packet allocates nine slots, resulting in an allocated time of 1.8 ms. In this case, for an error-free channel, the eLPRT protocol is able to support 49 nodes with 100% delivery ratio, which is almost twice the number of nodes supported by unslotted CSMA/CA mechanism in the best case (Figure 4). The maximum number of nodes supported by GTS scheme, if the limitation of seven nodes were removed, would be 14.

### 6.4. Transmission when the Beacon is Lost

The remaining results in this section take into account the occurrence of burst errors in the channel, through the use of the Gilbert-Elliot model, with the parameters presented in Section 4.

**Figure 6.**
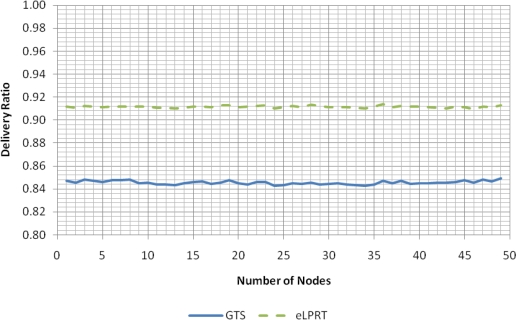
Delivery ratio of eLPRT and GTS, with *BER_bad_* = 10^−2^ and without retransmissions.

Figure 6 presents the delivery ratio as a function of the number of nodes in two cases. The first case, representing the normal functioning of the GTS scheme, considers that a node does not transmit its data packet when it misses the beacon at the beginning of the superframe. The second case uses the reallocation mechanism of the eLPRT protocol, enabling a node to transmit the data regardless of the reception of the beacon. In both cases, data packets are not retransmitted when affected by channel errors.

Given the packet lengths considered in the evaluation scenario, in the bad state (*BER_bad_* = 10^−2^), almost all (97.5%) data packets are corrupted by errors, while 76.5% of the beacons are affected. Since the delivery ratio of the eLPRT protocol depends only on the correct transmission of the data packet, and considering that the channel remains in the bad state 10% of the time, on average, the delivery ratio is slightly higher than 90% and independent from the number of nodes. The GTS scheme requires the correct transmission of both the beacon and the data packet, and therefore the delivery ratio is lower (around 84.5%).

### 6.5. Contention-Free Retransmissions

The results presented in this section concern the use of retransmissions, and consider two contention-free retransmission strategies. The first strategy (“RP after CAP”), adopted by the eLPRT protocol, places the Retransmission Period (RP) after the CAP and before the Normal Transmission Period (NTP). The second strategy (“RP before CAP”) places the RP after the beacon and before the CAP.

**Figure 7.**
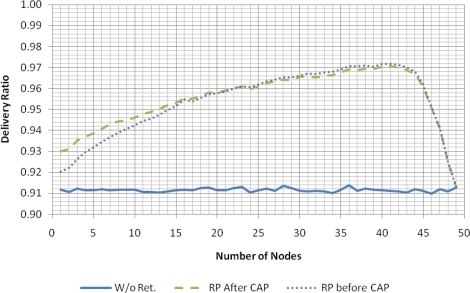
Delivery ratio of eLPRT, with *BER_bad_* = 10^−2^.

The delivery ratio results presented in Figure 7 use the same value of *BER_bad_* for both the beacon and the data packet. As the results show, the effectiveness of the retransmission mechanism with small number of nodes is low for both cases. The explanation is based on the fact that the allocation of slots for transmission of data packets starts from the end of the superframe and goes towards its beginning, as the number of nodes increases, which means that the transmissions of the first nodes are closely followed by the beacon of the next superframe. In a scenario with burst errors, there is a high probability that the bad state observed during the transmission of the data packets from these nodes extends into the reception of the beacon. When a node misses the beacon, it is unable to retransmit, since it does not have the information about the position of slots allocated for retransmission in the superframe. Therefore, burst errors tend to reduce the effectiveness of the retransmission mechanism, affecting more intensely data packets that are allocated near the following beacon. As the number of nodes in the network increases, the average distance between the data packets and the next beacon also increases, decreasing the probability that the beacon is affected by the bad state observed during the transmission of previous data packets.

When the BER that affects the beacon is high, it has a significant effect in the performance of both retransmission strategies. This effect is independent of the location of the retransmission period, since the retransmission information is not received for both strategies when the beacon is lost. Therefore, the delivery ratio for both strategies in this case is similar.

The increase of the output power of the base station transmitter enables decreasing the BER for the beacon frames since the bit error rate tends to decrease with the increase of the signal-to-noise ratio (SNR) [14]. This increment in the output power is not problematic in terms of energy consumption since, unlike the sensor nodes, the base station is not energy constrained. The base station’s output power can be raised, for example, through the use of a RF range extender such as the CC2591 [15], which provides a gain up to 22 dB.

**Figure 8.**
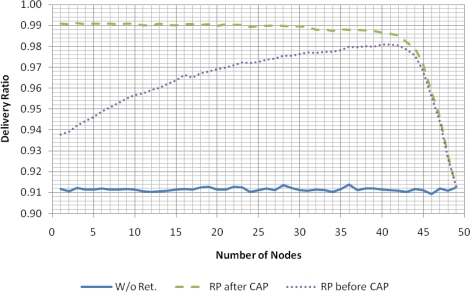
Delivery ratio of eLPRT with *BER_bad_* = 10^−2^ (data) and 10^−4^ (beacon).

Simulation results using the same conditions of the previous simulations, except for the *BER_bad_* relative to the reception of the beacon (was changed to 10^−4^, to account for an increase in the base station output power) are shown in Figure 8. Under these conditions, the delivery ratio for the “RP after CAP” strategy increases significantly, with particular relevance for small number of nodes. Regarding the “RP before CAP” strategy, no significant delivery ratio increase is observed. Since the retransmission is placed close to the failed transmission, the probability that burst errors affect both packets is higher in this last strategy.

For both cases, as the number of nodes approaches the capacity of the network, the space available for retransmissions in the superframe decreases and, consequently, the delivery ratio approaches the value without retransmissions.

**Figure 9.**
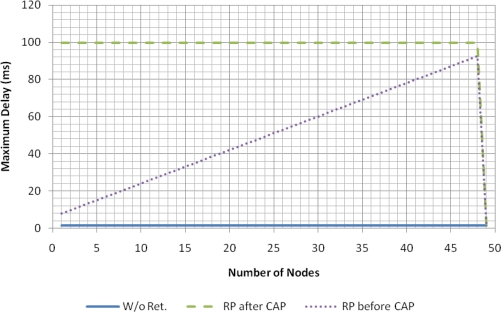
Maximum delay of eLPRT with *BER_bad_* = 10^−2^ (data) and 10^−4^ (beacon).

The larger separation between the original data packet and the scheduled retransmission in the “RP after CAP” strategy has the benefit of increased robustness against burst errors. Although the average delay with this strategy is higher, for real-time applications, the provision of bounded delay is more important than the reduction of the average delay. As Figure 9 shows, for the “RP after CAP” strategy, the maximum delay suffered by the data packets is bounded by the superframe period (100 ms).

**Figure 10.**
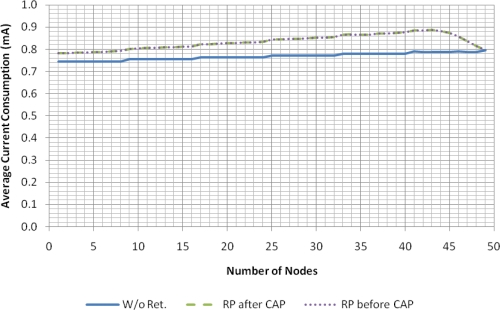
Current consumption of eLPRT with *BER_bad_* = 10^−2^ (data) and 10^−4^ (beacon).

Figure 10 shows the average current consumption (per sensor node) of the eLPRT protocol using the parameters of the CC2430 included in Section 4. The consumption for both retransmission strategies is equal, and increases slightly with the number of nodes, since the size of the beacon increases due to the ACK bitmap field and the allocations for retransmissions. Neverthelesss, the increase in the current consumption due to retransmissions is small, being largely compensated by the benefit of increased delivery ratio. As an example, the current consumption with 25 nodes is 0.77 mA without retransmissions and 0.84 mA with retransmissions due to channel errors (9% more). Experimental results concerning the use of the frequency hopping mechanism in combination with the contention-free retransmission mechanism are provided in the next section.

## 7. System Prototype

The developed wireless motion capture system is composed by three main components: a personal computer (PC), a base station and one wireless sensor node for each segment of the body requiring monitoring.

The main component of the wireless sensor node is the CC2430 [10], from Texas Instruments, a SoC (System on Chip) that integrates an 8051 based microcontroller and an IEEE 802.15.4 compliant transceiver in the same chip. A printed circuit antenna compatible with CC2430 radio was also implemented [16], effectively reducing the size of the sensor node and making it less obtrusive. The sensor node is powered by a 300 mAh rechargeable lithium-ion battery.

**Figure 11.**
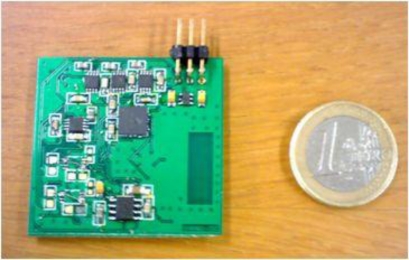
Wireless sensor node prototype board.

Each sensor node also contains a 3-axis accelerometer and a 3-axis magnetometer, that are used to obtain the pitch, roll and yaw angles through a process described in patent WO 2008/018810 A2 [17]. Both the gravitational force and the Earth’s magnetic field are used to detect the angles of the segments. The former is used to detect inclination while the later is used to measure the rotation of the body about the axis perpendicular to the gravity field. Fifth order low pass elliptical filters are used to minimize noise from sensor readings. A DAC (Digital-to-Analog Converter) with adjustable current enables calibration of the magnetic sensors, which are sensitive to magnetic field variations. Each sensor node is able to provide resolution around 1 degree. Figure 11 shows the sensor node prototype board.

The implementation of the base station is based on the SmartRF04EB and CC2430EM boards provided by the CC2430 Development Kit from Texas Instruments. The CC2591 RF range extender can be used to increse the base station’s output power. The base station is powered by the PC through a USB cable.

The PC application receives the data acquired from the sensor nodes, calculates the angles of the segments of the user’s body, generates a 3D model compliant with the BVH (Biovision Hierarchy) file format [18] and displays the movement of the user’s body in real-time.

### 7.1. Protocol Implementation

**Figure 12.**
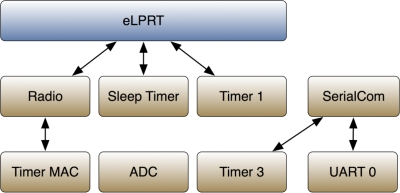
System software modules.

Figure 12 presents the relation between the software modules used to control the hardware and the software modules that implement the eLPRT protocol and the serial port communications. The SerialCom and related modules are only implemented in the base station, while the ADC module is only implemented in the sensor nodes.

The Timer MAC module generates time intervals required by the Radio module. This is a 16-bit timer with adjustable period and is used to execute backoff periods and to manage the timeout for reception of the acknowledgment frame in messages sent with the acknowledgment request active.

The Sleep Timer’s main function is to generate time intervals between events, during which the radio and microprocessor can be turned off in order to save energy. It is a 24-bit timer that uses a crystal oscillator as time source, counting uninterruptedly after a system reset.

Timer 1 is a 16-bit timer with three independent channels. Its operating frequency is derived from the main system clock (32 MHz) and can be divided by 8, 32 or 128. This module is used in the eLPRT implementation to generate time intervals associated to the protocol, such as the superframe period and the duration of the access periods, as well as to account for time elapsed between events.

Among the 8-bit timers available in CC2430, only timer 3 was used. This timer is used in the serial communications to control time intervals between transmission and reception of acknowledgment messages.

The UART 0 module controls the configurations regarding the serial port communications, namely the baud rate, number of data bits, number of stop bits and parity. In order to avoid the serial port becoming a bottleneck of the system, the baud rate of the UART must be greater than the data rate of the radio interface (250 kbps). For this reason, the baud rate was set to 460,800 Bd. The SerialCom module builds its functionalities on top of the UART 0 module and allows full duplex communication in the serial connection.

### 7.2. Experimental Results

**Figure 13.**
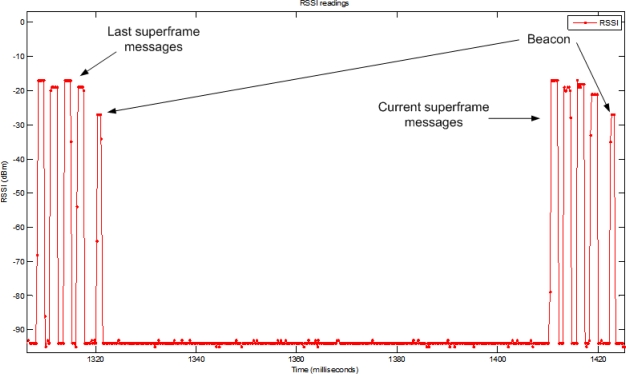
Transmissions of four sensor nodes in the NTP.

In order to validate the implementation of the MAC protocol, a test tool was created. This tool runs in a separate CC2430 module connected to a PC, continuously collecting received signal strength indicator (RSSI) samples at the selected channel. Each sample is obtained every 128 μs, by reading the RSSI register of the radio transceiver, along with the first byte of the sleep timer counter, which represents the axis of time. These values are sent through the serial connection to a PC application, which plots a graph with the RSSI as a function of time. Figure 13 shows the RSSI values with four allocations in the NTP. All transmissions are made with an output power of 0 dBm.

**Figure 14.**
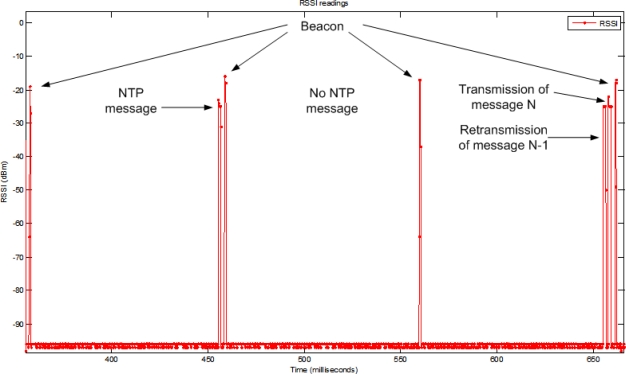
Example of operation of the contention-free retransmission mechanism.

Figure 14 exemplifies the operation of the contention-free retransmission mechanism of the eLPRT protocol. The first superframe shows the transmission of a data packet (message) in the allocated slots in the NTP. In the second superframe, the sensor node does not transmit its associated data packet on purpose, to simulate the loss of the packet; consequently, the base station automatically assigns a RP allocation to the node in the next superframe. The packet is then retransmitted before the transmission of respective data packet of that superframe.

The next results show the effectiveness of the frequency hopping mechanism implemented by the eLPRT protocol. The experimental tests were performed with one base station and one sensor node inside a RF shielded anechoic chamber with 2.91 m (length) by 2.06 m (width) by 2.06 m (height). The base station and the node were placed in the center of the chamber at a distance of 1.67 m from each other and at a height of 1.1 m from the ground. The output power of both the base station and the sensor node was set to 0 dBm and the level of the signal received from the sensor node by the base station was −47 dBm. The IEEE 802.15.4 channel 22 was used in the test without frequency hopping.

**Figure 15.**
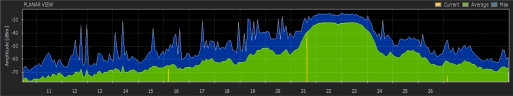
Spectrum of the 802.11g interference.

The interference source consisted of a file transfer between two laptop computers using the IEEE 802.11g standard [19] at channel 11, the DCF (Distributed Coordination Function), ad-hoc mode and bit rate of 54 Mbps. Figure 15 shows the spectrum of the IEEE 802.11g interference (the numbers displayed in the horizontal axis correspond to the IEEE 802.15.4 channels) measured using the Wi-Spy 2.4x [20] spectrum analyzer. The transmitter laptop was placed 40 cm to the side of the sensor node and the receiver laptop was placed 40 cm to the same side of the base station. Both laptops were placed at a height of 20 cm from the ground. The strength of the 802.11 interference measured at the base station was in the range between −35 dBm and −40 dBm. The generated interference was almost continuous, except for the backoff periods and interframe spaces associated to the IEEE 802.11 DCF protocol.

The experimental tests used the traffic and network parameters indicated in Section 4, as well as all features of the eLPRT protocol. Each test was executed during 30 minutes, which corresponds to 18,000 superframes. The results are summarized in Table 4. The hopping sequence is given by Equation (1) and depends on the channel jump (*n_j_*) parameter. The delivery ratio (DR) without retransmissions corresponds to the percentage of data packets transmitted in the NTP that are successfully delivered to the base station. The DR with retransmissions includes also the packets successfully retransmitted in the RP of the next superframe using the contention-free retransmission mechanism. The last row of the table shows the percentage of lost data packets that the retransmission mechanism was able to recover.

In the first test (*Ch* = 22), the frequency hopping mechanism was not used and the eLPRT network remained in channel 22 of IEEE 802.15.4 all the time. The DR without retransmissions was low (61.2%) because, as Figure 15 shows, this channel overlaps with channel 11 of IEEE 802.11. The retransmission mechanism was able to recover only 43% of the lost packets, since the beacon and the retransmitted packet were also subject to the interference.

The three cases where the frequency hopping mechanism was used presented similar results for the DR without retransmissions, ranging from 89.6% to 92.5%. Results are better than in the previous case since only 25% of the superframes (corresponding to four of the 16 IEEE 802.15.4 channels) were significantly affected by the 802.11 interference. The combination of the contention-free retransmissions mechanism with the frequency hopping mechanism makes the lost transmissions and the corresponding retransmissions occur in different IEEE 802.15.4 channels. When a transmission is lost due to interference from a IEEE 802.11 channel, a jump of five IEEE 802.15.4 channels is always enough to allow the retransmission to escape from that interference. Therefore, as the results show, with *n_j_* = 5, the retransmissions where able to recover all lost data packets, allowing the DR with retransmissions to reach 100%. Retransmissions are less efficient with jumps of one (*n_j_* = 1) or three (*n_j_* = 3) channels, because such jumps are not long enough to allow the retransmissions to escape interference from an IEEE 802.11 channel all the time.

## 8. Conclusions

The proposed eLPRT protocol introduces several features designed to enhance the performance in comparison to the IEEE 802.15.4 schemes. Results showed that, in the considered evaluation scenario, the eLPRT protocol is able to support the traffic of much more nodes than the unslotted CSMA/CA scheme of IEEE 802.15.4 while consuming significantly less energy.

Regarding the comparison with the GTS scheme of the IEEE 802.15.4, the fine granularity of the slots provided by the eLPRT protocol eliminates the wasted bandwidth due to the underutilization of the GTS slots. The provision of the Allocation Identifier (AID) allows the eLPRT allocation descriptor to maintain the same size of the GTS descriptor. The ACK bitmap mechanism eliminates the overhead associated with the transmission of an ACK frame for each uplink data packet, increasing bandwidth efficiency. The reallocation mechanism provided by the eLPRT protocol allows nodes to transmit data in the allocated slots even when the beacon of that superframe is lost, increasing the reliability against channel errors.

The contention-free retransmission mechanism provided by the eLPRT protocol can be used to increase the delivery ratio, with only a small increment in the node energy consumption. Its effectiveness increases if a mechanism to increase the robustness of the beacon is also provided.

The frequency hopping mechanism, in combination with the retransmission mechanism, enables the exploration of frequency diversity to recover from transmission errors caused by interference from an IEEE 802.11 network. The effectiveness is higher if the channel jump is long enough to guarantee that the interference does not affect two consecutive superframes.

## Figures and Tables

**Table 1.** Unslotted CSMA/CA parameters.

**Parameter**	**Description**	**Value**
*macMinBE*	The minimum value of the backoff exponent	[0–3], default = 3
*aUnitBackoffPeriod*	The length of the backoff period.	20 symbols
*aMaxBE*	The maximum value of the backoff exponent	5
*macMaxCSMAbackoffs*	The maximum number of backoff periods	[0–5], default = 4

**Table 2.** Parameters of the Gilbert-Elliot model.

**Parameter**	**Value**
*BER_bad_*	10^−2^
*BER_good_*	0
*T_bad_*	20 ms
*T_good_*	180 ms

**Table 3.** Number of samples per packet with different superframe periods.

***T_SF_* = 61.44 ms**			***T_SF_* = 122.88 ms**			***T_SF_* = 100 ms**		

**Scheduled time**	**Number of samples**		**Scheduled time**	**Number of samples**		**Scheduled time**	**Number of samples**	
	***f_s_* = 10 Hz**	***f_s_* = 30 Hz**		***f_s_* = 10 Hz**	***f_s_* = 30 Hz**		***f_s_* = 10 Hz**	***f_s_* = 30 Hz**

0	1	1	0	1	1	0	1	1
61.44	0	1	122.88	1	3	100	1	3
122.88	1	2	245.76	1	4	200	1	3
184.32	0	2	368.64	1	4	300	1	3
245.76	1	2	491.52	1	3	400	1	3
307.20	1	2	614.40	2	4	500	1	3
368.64	0	2	737.28	1	4	600	1	3
430.08	1	1	860.16	1	3	700	1	3

**Table 4.** Experimental results concerning the use of the frequency hopping mechanism.

	***Ch* = 22**	***n_j_* = 1**	***n_j_* = 3**	***n_j_* = 5**
**DR without retransmissions**	61.2%	91.8%	92.5%	89.6%
**DR with retransmissions**	77.9%	97.7%	99.8%	100%
**Recovered packets**	43.0%	72.0%	97.3%	100%

## References

[1] (2006). IEEE 802.15.4 Standard. Part 15.4: Wireless Medium Access Control (MAC) and Physical Layer (PHY) Specifications for Low-Rate Wireless Personal Area Networks (LR-WPANs).

[2] Buratti C, Conti A, Dardari D, Verdone R (2009). An Overview on Wireless Sensor Networks Technology and Evolution. Sensors.

[3] Song JK, Ryoo JD, Kim SC, Kim JW, Kim HY, Mah PS A Dynamic GTS Allocation Algorithm in IEEE 802.15.4 for QoS guaranteed Real-time Applications.

[4] Koubaa A, Alves M, Tovar E i-GAME: An Implicit GTS Allocation Mechanism in IEEE 802.15.4 for Time-Sensitive Wireless Sensor Networks.

[5] Cheng L, Bourgeois AG, Zhang X A New GTS Allocation Scheme for IEEE 802.15.4 Networks with Improved Bandwidth Utilization.

[6] Rocha LA, Correia JH Wearable Sensor Network for Body Kinematics Monitoring.

[7] Rocha LA, Afonso JA, Mendes PM, Correia JH A Body Sensor Network for E-Textiles Integration.

[8] Paksuniemi M, Sorvoja H, Alasaarela E, Myllylä R Wireless Sensor and Data Transmission Needs and Technologies for Patient Monitoring in the Operating Room and Intensive Care Unit.

[9] Afonso JA, Rocha LA, Silva HR, Correia JH MAC Protocol for Low-Power Real-Time Wireless Sensing and Actuation.

[10] (2007). CC2430 Data Sheet (Rev. 2.1).

[11] Varga A The OMNeT++ Discrete Event Simulation System.

[12] Ebert J, Willig A (1999). A Gilbert-Elliot Bit Error Model and the Efficient Use in Packet Level Simulation.

[13] Willig A (2008). Recent and Emerging Topics in Wireless Industrial Communications: A Selection. IEEE Trans. Indust. Inform.

[14] Zuniga M, Krishnamachari B (2004). Analyzing the Transitional Region in Low Power Wireless Links.

[15] (2009). Using CC2591 Front End with CC2530/1.

[16] (2008). Small Size 2.4 GHz PCB Antenna.

[17] Afonso JA, Correia JH, Silva HR, Rocha LA (2008). Body Kinetics Monitoring System. International Patent WO/2008/018810A2.

[18] Lander J (1998). Working with Motion Capture File Formats. Game Developer Magazine.

[19] (2007). IEEE Standard 802.11-2007. Part 11: Wireless LAN Medium Access Control (MAC) and Physical Layer (PHY) Specifications.

[20] Wi-Spy www.metageek.net/products/wi-spy/.

